# Successful malaria elimination in the Ecuador–Peru border region: epidemiology and lessons learned

**DOI:** 10.1186/s12936-016-1630-x

**Published:** 2016-11-28

**Authors:** Lyndsay K. Krisher, Jesse Krisher, Mariano Ambuludi, Ana Arichabala, Efrain Beltrán-Ayala, Patricia Navarrete, Tania Ordoñez, Mark E. Polhemus, Fernando Quintana, Rosemary Rochford, Mercy Silva, Juan Bazo, Anna M. Stewart-Ibarra

**Affiliations:** 1 Center for Health, Work & Environment, Colorado School of Public Health, University of Colorado Denver, Aurora, CO USA; 2 Center for Global Health and Translational Science, State University of New York Upstate Medical University, 505 Irving Ave., Syracuse, NY 13210 USA; 3 Ministerio de Salud Pública, Machala, El Oro, Ecuador; 4 Facultad de Medicina, Universidad Técnica de Machala, Machala, El Oro, Ecuador; 5 Dirección Regional de Salud Tumbes, Ministerio de Salud de Peru, Tumbes, Peru; 6 Department of Environmental and Occupational Health, Colorado School of Public Health, University of Colorado Denver, Aurora, CO USA; 7 Red Cross/Red Crescent Climate Centre, Lima, Peru

**Keywords:** Ecuador, Peru, Malaria, Elimination, Border region, Vector control, *Plasmodium falciparum*, *Plasmodium vivax*, Binational collaboration

## Abstract

**Background:**

In recent years, malaria (*Plasmodium vivax* and *Plasmodium falciparum*) has been successfully controlled in the Ecuador–Peru coastal border region. The aim of this study was to document this control effort and to identify the best practices and lessons learned that are applicable to malaria control and to other vector-borne diseases. A proximal outcome evaluation was conducted of the robust elimination programme in El Oro Province, Ecuador, and the Tumbes Region, Peru. Data collection efforts included a series of workshops with local public health experts who played central roles in the elimination effort, review of epidemiological records from Ministries of Health, and a review of national policy documents. Key programmatic and external factors are identified that determined the success of this eradication effort.

**Case description:**

From the mid 1980s until the early 2000s, the region experienced a surge in malaria transmission, which experts attributed to a combination of ineffective anti-malarial treatment, social-ecological factors (e.g., El Niño, increasing rice farming, construction of a reservoir), and political factors (e.g., reduction in resources and changes in management). In response to the malaria crisis, local public health practitioners from El Oro and Tumbes joined together in the mid-1990s to forge an unofficial binational collaboration for malaria control. Over the next 20 years, they effectively eradicated malaria in the region, by strengthening surveillance and treatment strategies, sharing of resources, operational research to inform policy, and novel interventions.

**Discussion and evaluation:**

The binational collaboration at the operational level was the fundamental component of the successful malaria elimination programme. This unique relationship created a trusting, open environment that allowed for flexibility, rapid response, innovation and resilience in times of crisis, and ultimately a sustainable control programme. Strong community involvement, an extensive microscopy network and ongoing epidemiologic investigations at the local level were also identified as crucial programmatic strategies.

**Conclusion:**

The results of this study provide key principles of a successful malaria elimination programme that can inform the next generation of public health professionals in the region, and serve as a guide to ongoing and future control efforts of other emerging vector borne diseases globally.

**Electronic supplementary material:**

The online version of this article (doi:10.1186/s12936-016-1630-x) contains supplementary material, which is available to authorized users.

## Background

According to the Pan American Health Organization (PAHO), 145 million people in 21 countries of the Americas are at risk of contracting malaria [1]. However, significant investments in malaria control programmes have resulted in major reductions in transmission. In 2012, 149,000 cases were confirmed positive, resulting in 108 deaths, representing a 60% decline in case incidence and 72% decline in mortality since 2000 in the Americas [1].

The Ecuador–Peru coastal border region is an example of successful malaria control; this region is historically endemic for *Plasmodium vivax* and *Plasmodium falciparum.* El Oro, the southernmost coastal province of Ecuador, has been free of local malaria transmission since 2011; in 2014, only one imported case was registered [2]. At a national-level, the burden of malaria in Ecuador declined by 99%, from 106,641 annual cases in 2001 to 558 cases in 2012. In 2012, the country was recognized as one of three “Malaria Champions of the Americas,” and progressed from the “control” to the “pre-elimination” phase of malaria management, based on World Health Organization (WHO) criteria. In Tumbes, the northern-most coastal province of Peru, the last case of locally transmitted malaria was reported in 2012 (MoH Tumbes pers. comm. 2014*)*. As of 2012 Peru was in the “control” phase of malaria management, with a 64% decrease in malaria cases since 2000, placing the country on track to achieve the United Nations (UN) Millennium Development Goal of a 75% decrease by 2015, which Ecuador has achieved [3].

This paper describes the epidemiology, social-ecological conditions, strategies and actors who contributed to the successful reduction in malaria transmission in the Ecuador–Peru border region. Due to the important role of local climate in both seasonal and interannual malaria dynamics, a detailed time series of climate conditions, El Niño events, and malaria epidemiology are included. In 2014, key personnel from El Oro and Tumbes convened twice to generate a timeline of events and to identify the best practices and lessons learned that apply to malaria control and other vector-borne diseases (see Additional file 1: Table S1 for names and roles, meeting minutes available upon request). The findings presented here reflect the consensus from those meetings, as was done in similar studies [4, 5]. Findings are supported by a review of epidemiologic records provided by the Ecuadorian and Peruvian Ministries of Health (MoH), climate information provided by the National Institute of Meteorology and Hydrology of Ecuador (INAMHI) and the National Meteorology and Hydrology Service of Peru (SENAMHI), national policy documentation, and an exhaustive literature review [6] in Google Scholar, PubMed, the WHO Library (WHOLIS), the Global Fund Library and PAHO, using the search terms “ “Ecuador” or “Peru” or “Latin America” and “malaria” or “paludismo” and “elimination” or “prevention” or “control” or “treatment” or “diagnosis” or “*plasmodium falciparum*” or “*plasmodium vivax*.” Published academic papers and grey literature in both English and Spanish were included in the analysis. This study contributes to an ongoing collaboration with the Ecuadorian Ministry of Health to strengthen the surveillance of endemic and emerging febrile vector-borne diseases in the region.

## Case description

### Data sources

Monthly cases of malaria from 1990 to 2012 were provided by the MoH in Ecuador and Peru. Malaria is a mandatory notifiable disease in both countries (case reporting described below). Incidence was calculated using population data from the national censuses in Ecuador conducted by the Instituto Nacional de Estadística y Censos (INEC) (1990, 2001, 2010 [7]) and data from Peru provided by the MoH Dirección Regional de Salud in Tumbes [8]. Populations in years between censuses were estimated assuming linear growth.

Local daily weather data (rainfall, mean/maximum/minimum temperature) were provided by the Granja Santa Ines weather station located in Machala, El Oro, Ecuador (3°17′26″ S, 79°54′5″ W, 10 m.a.s.l.) and the Puerto Pizarro station located in Tumbes, Peru (3° 53′ S, 80° 35′) operated by INAMHI and SENAMHI, respectively. The Oceanic Niño Index (ONI) (ERSST.v4) was provided by The National Oceanic and Atmospheric Administration (NOAA) Climate Prediction Center of NOAA/National Weather Service [9]. Average monthly values and monthly anomalies in malaria incidence, total monthly rainfall, and air temperature were calculated using R [10].

### Study site

**Fig. 1:**
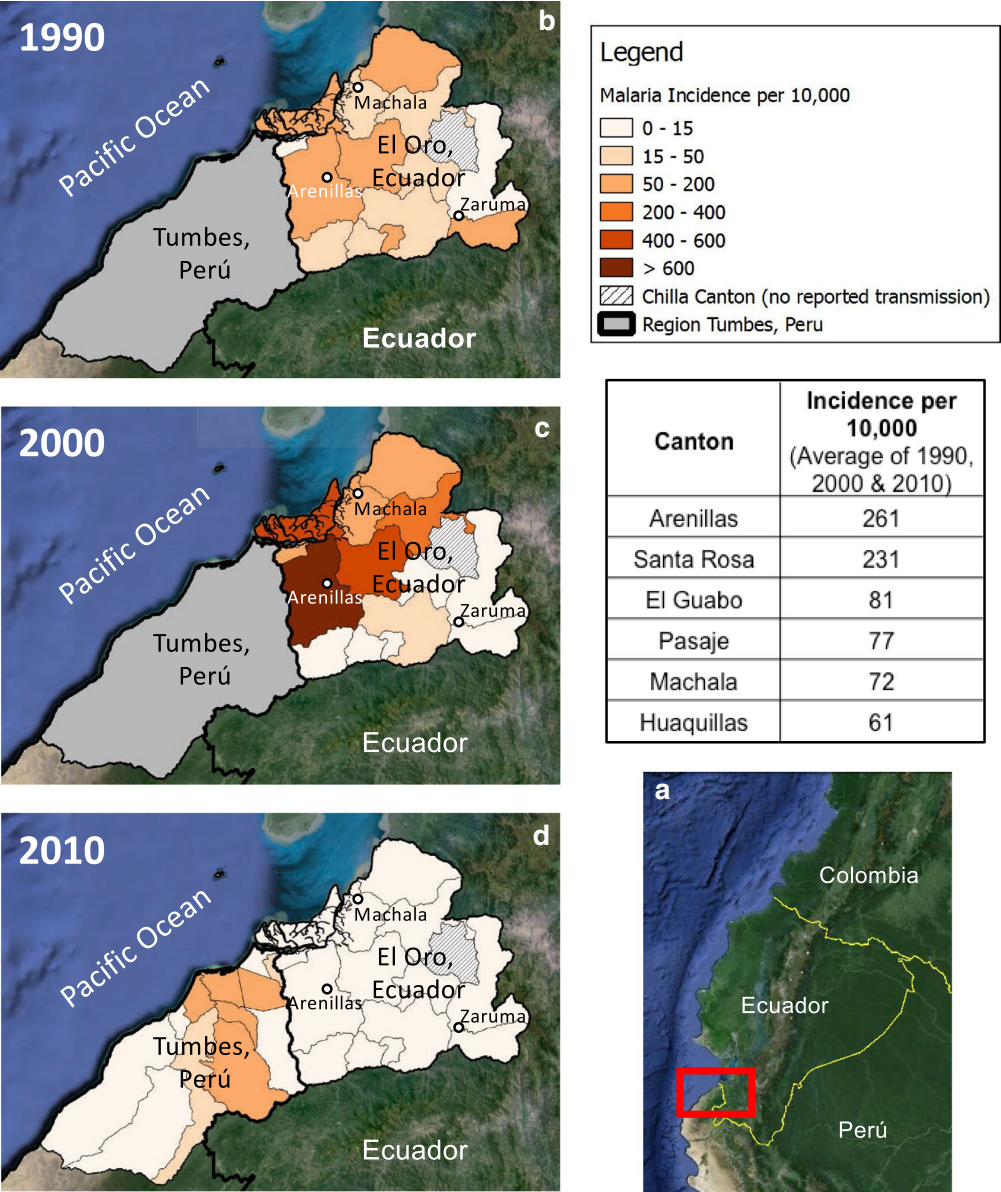
**a** Coastal border region between Ecuador and Peru (El Oro Province and Region of Tumbes) (Google Earth, 2013). **b**, **c** and **d** exhibit the incidence of malaria by canton (El Oro) or district (Tumbes) in 1990, 2000, and 2010 (base map from Google Earth, 2013). Incidence is shown in El Oro for all three periods, and in Tumbes for the year 2010, when data were available

**Fig. 2:**
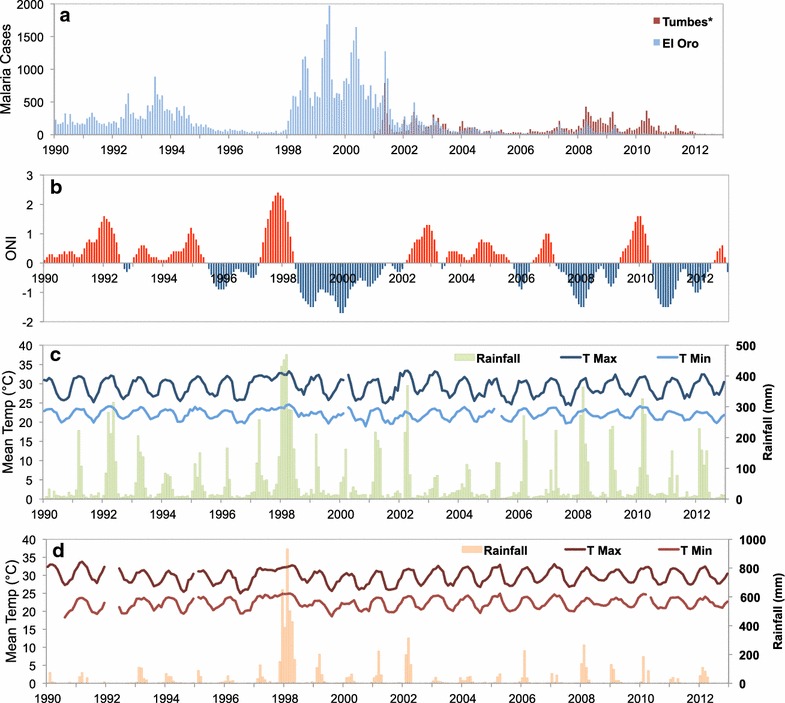
Time series of monthly malaria cases and climate for El Oro and Tumbes: **a** monthly malaria cases (*no monthly cases available for Tumbes before 2001), **b** Oceanic Niño Index, 3-month running mean anomalies in sea surface temperature in the Niño 3.4 region, **c** El Oro mean monthly temperature (maximum and minimum) and rainfall (mm), **d** Tumbes mean monthly temperature (maximum and minimum) and rainfall (mm)

**Fig. 3:**
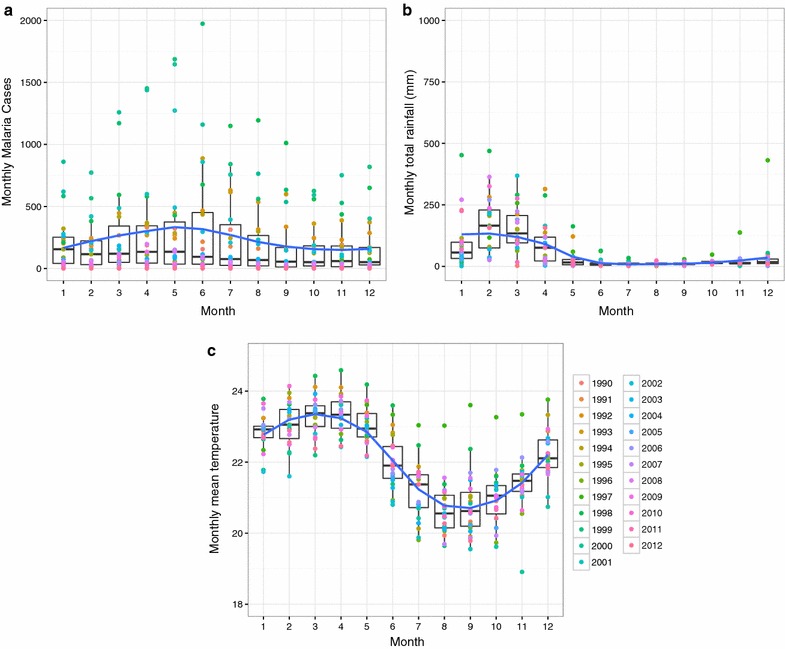
Seasonality of malaria and climate in El Oro (1990–2012). *Box plots* shown with a loess smoothing function for **a** monthly malaria cases, **b** total monthly rainfall, and **c** mean minimum air temperature

**Fig. 4:**
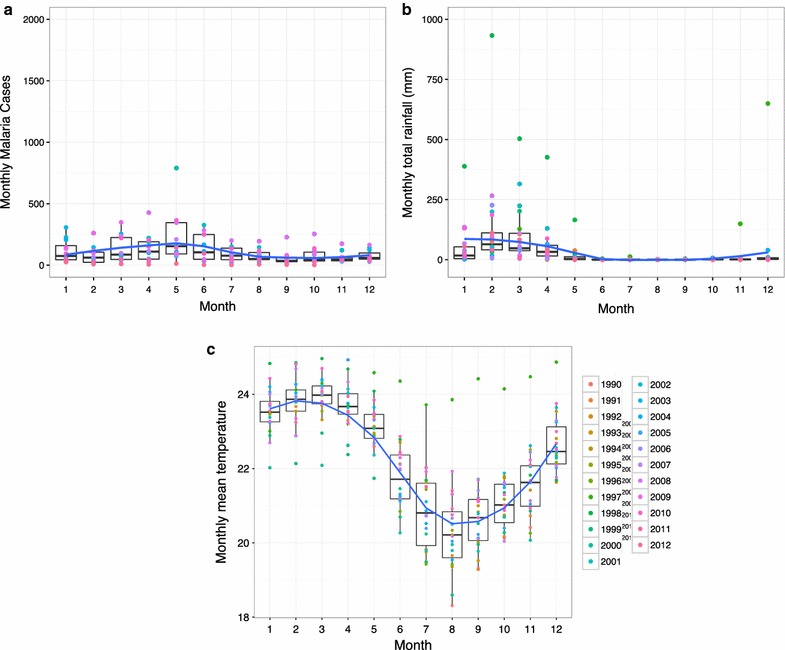
Seasonality of malaria (2001–2012) and climate in Tumbes (1990–2012). *Box plots* shown with a loess smoothing function for **a** monthly malaria cases, **b** total monthly rainfall, and **c** mean minimum air temperature

The El Oro Province of Ecuador (population 600,659) and the bordering Tumbes Region of Peru (population 200,306) are coastal, tropical agricultural provinces (latitude: 3°5′45.20″S–4°11′3.06″S, longitude:79°43′10.92″W–80°50′37.96″W). The regions are linked by the Pan American highway, resulting in significant cross-border migration, especially from Peru into Ecuador [11]. Transmission in El Oro Province has historically been concentrated in the tropical coastal lowlands, in the cantons (counties) of Arenillas and Santa Rosa (Fig. 1). The northern coastal border region of Peru is the second most important region for malaria transmission in the country, following the Amazon region. Transmission in the Tumbes region has historically been concentrated near the border with Ecuador in the districts of Aguas Verdes and Zarumilla. Malaria transmission is highly seasonal, peaking in May at the end of the hot rainy season, when temperatures and rainfall are optimal for disease transmission (Figs. 2, 3, 4). The climate in Tumbes is more arid than El Oro, with the capital city of Tumbes receiving half the annual rainfall of the city of Machala.

Malaria in this region is transmitted primarily by the *Anopheles albimanus* and *Anopheles pseudopunctipennis* mosquitoes (M. Silva, former director of entomology, SNEM El Oro, *pers. comm.*). *Anopheles albimanus* larvae can survive in slow-moving fresh or brackish water [12], such as the mangrove swamps that characterize this coastal border region. Natural and man-made environments (e.g. rice fields) with sun exposure and some clear water are suitable for these vectors [13]. *Anopheles pseudopunctipennis*, like *An. albimanus*, prefer environments that are sunlit and include clear freshwater pools and streams. Both species are characterized by evening and nighttime biting behaviours [12].

### Malaria management

Malaria management in Ecuador was overseen historically by the National Vector Control Service (SNEM) of the MoH [14], which was established by external agencies (e.g., Rockefeller Foundation and others) to combat malaria and yellow fever. The programme ran for many decades, with a top-down militaristic approach focused on the use of DDT and rigorous case management, which the group of experts reported to be highly effective. In 1991, the MoH officially took over management of SNEM in Ecuador, which continued to operate as a vertical semi-autonomous organization. In 2015, SNEM was dissolved and vector control activities were decentralized and integrated into local MoH epidemiology and surveillance programmes.

Peru similarly transitioned from external management and funding of their malaria programme, to management and increasing funding by the MoH in the 1990s. Today the malaria control programme is decentralized and integrated into the MoH. Diagnostics are managed principally by the National System of Public Health Laboratories (*Sistema de la Red Nacional de Laboratorios*) under the National Institute of Health (*Instituto Nacional de Salud*). Vector control and surveillance in Peru are managed by the entomology unit of the Environmental Health division of the MoH.

### The malaria crisis

**Fig. 5:**
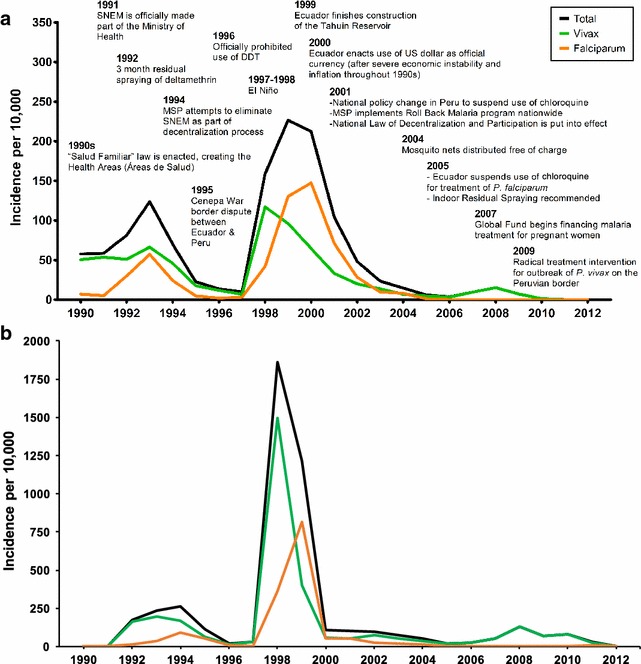
**a** Timeline of malaria incidence in El Oro, Ecuador, (cases per 10,000 people per year) and events affecting malaria control in the Ecuador–Peru border region from 1990 to 2012. Events occurred in Ecuador except where otherwise specified. **b** Time series of malaria incidence in the Tumbes Region, Peru (cases per 10,000 people per year) from 1990 to 2012

The group of experts reported that prior to the 1980s, both Ecuador and Peru were able to successfully reduce malaria transmission in the border region. However, from the mid 1980s until the early 2000s, the region experienced a surge in malaria transmission. In El Oro, 33,602 cases of *P. vivax* and 28,509 cases of *P. falciparum* were reported from 1990 to 2012. The average incidence was 30.28 cases of *P. vivax* and 24.89 cases of *P. falciparum* per 10,000 people per year (Fig. 5 **)**. During the same period (1990–2012) in the Tumbes Region of Peru, 57,783 cases of *P. vivax* and 27,822 of *P. falciparum* were reported, resulting in an average yearly incidence of 139.03 and 67.51 per 10,000 people, respectively. Malaria incidence in the Tumbes Region peaked at 1858 cases per 10,000 people in 1998, and in El Oro Province, transmission peaked at 226.5 cases per 10,000 people in 1999. The experts attributed the surge in malaria transmission to a combination of ineffective anti-malarial treatment, social-ecological and political factors.

Difficult clinical management and drug resistance were identified as among the most important factors leading to the high burden of disease. During the 1990s, teams in El Oro and Tumbes found evidence of drug resistance to chloroquine for *P. falciparum*. Experts also reported that *P. vivax* was notoriously difficult to treat; patients were required to complete a long course of anti-malarial medication (14 days of once daily primaquine, 15 mg for adults, 7.5 mg. for children). Patients would often stop taking the medication after a few days when they started feeling better, leading to relapse.

Key social-ecological factors included extreme climate events triggered by the El Niño Southern Oscillation, building of a reservoir, and expansion of agricultural areas. Climate played an important role in the timing of malaria epidemics in the region, with peaks in malaria transmission following strong El Niño events (1982–1983 in Ecuador, 1998 in Tumbes (Fig. 5), and 1999 in El Oro) and a decline in malaria following strong La Niña events (1998–2001) (Fig. 2). El Niño events are associated with warmer air temperatures and increased rainfall in the region, optimal conditions for the development of the mosquito vector and the parasite. Experts also hypothesized that the 1998–1999 malaria epidemic was exacerbated by changes in microclimate, related to the construction of a large reservoir built near the border in 1999 (Tahuín Reservoir, city of Arenillas). After the reservoir was built, they observed a shift from seasonal to year-round transmission of malaria for the first time in the region; however, no formal studies were conducted to determine the effect of the reservoir. Experts also reported that increased rice production in Tumbes had increased the risk of malaria transmission, by expanding larval habitats for *Anopheles* mosquitoes.

Key political factors included reductions in resources and changes in the management structure of malaria control programmes. Successful control of malaria prior to the 1980s resulted in a decline in external funding. National governments took over management of the vector control programmes in the 1990s, leading to instability in management and resourcing. In 1994 the Ecuadorian government tried to eliminate SNEM in an attempt to decentralize MoH activities 15]. However, experts reported that the decentralization was ineffective, due to limited allocation of personnel and other resources, and SNEM persisted as a centralized body in Ecuador. Resource limitations led both programmes to reduce the number of field personnel and to discontinue environmental interventions, such as mosquito surveillance and control. Experts reported that the MoH began using insecticides at lower concentrations to save resources, and IRS interventions were implemented only in households with confirmed malaria cases. The group reported that the weakening of their programmes resulted in a deterioration of their ability to serve the communities and the erosion of public trust. The decline in institutional capacity coincided with the 1982–1983 El Niño event, resulting in severe malaria outbreaks and resurgence in endemic transmission over the next 15 years (Fig. 5).

### Binational collaboration

In response to the malaria crisis, local leaders from El Oro and Tumbes joined together in the mid 1990s to forge an unofficial binational collaboration for malaria control. This natural partnership emerged to address the shared challenges of managing a malaria epidemic in a transient border population. Over the next 20 years, local public health leaders from both countries worked closely to strengthen surveillance and response strategies, through sharing of resources, operational research to inform policy, and novel interventions to halt transmission. The close relationships and strong, stable leadership at the local operational level were keys to the success of the malaria control programmes.

### Co-learning through operational research

Through this binational collaboration, experts reported that public health practitioners in El Oro and Tumbes created an environment where they were able to share and adapt local malaria control strategies and lessons, resulting in changes in national policy. In the early 1990s, the Malaria Control Programme team in Tumbes recognized that patients with *P*. *falciparum* were responding poorly to chloroquine, the standard treatment at the time. They conducted epidemiological studies and found evidence of drug resistance to chloroquine for treatment of *P. falciparum*. As a result, the Tumbes region discontinued the use of chloroquine in 1993 in favor of sulfadoxine-pyrimethamine, despite Peruvian national recommendations to the contrary (MoH Tumbes, pers. comm.). It was not until 2001 that Peru officially changed its national recommendations to discontinue the use of chloroquine due to drug-resistance, evidence originally provided by the local team. The team also conducted studies to evaluate the effectiveness of changing the treatment for *P. vivax* from one tablet of primaquine daily for 14 days to 2 tablets daily for 7 days. They found that patients were more likely to complete the course of medication and to clear the parasite, effectively cutting transmission. Based on the experience in Tumbes, the SNEM team in El Oro repeated these studies following the 1998–1999 malaria epidemic, with direction and technical assistance from The Amazon Network for the Surveillance of Antimalarial Drug Resistance (in Spanish, RAVREDA, or Red Amazónica de Vigilancia de la Resistencia de las Drogas Antimaláricas). The binational team sent their report to the Ecuadorian national government, and in 2006 the MoH officially recognized the findings and adopted the recommended change to artemisinin-based combination therapy (ACT) and the new treatment schedule for *P. vivax* [16]. Experts attributed the rapid decline in malaria transmission in the region to these changes in clinical management. Following these successes, SNEM leaders from the region played a key role in the development and publication of a practical guide to conducting drug efficacy studies together with PAHO, the U.S. Centers for Disease Control (CDC), and USAID [17]. These resistance studies demonstrated that co-learning and local operational research projects can have a significant impact on national public health policy.

### Sharing of resources to build resilience

**Fig. 6:**
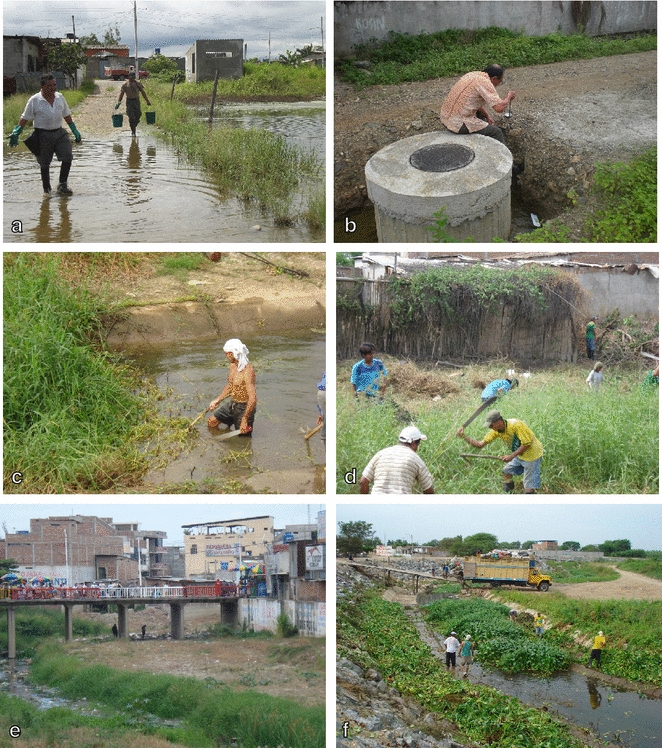
Photography of SNEM personnel in collaborative work on the border (SNEM archives-El Oro). **a** Treating flooded area with diesel. **b** Characterization of mosquito breeding site. **c**–**f**. Community-led, SNEM supported cleanup (“minga”) of the International Canal between El Oro and Tumbes in 2008. SNEM in Tumbes reported a 40% decline in malaria cases following the extensive cleaning of the canal (MINSA 2008. http://www.minsa.gob.pe/portada/prensa/nota_completa.asp?nota=6214)

The group of experts reported that one of the key components of the collaboration was the sharing of information, medication, insecticides, and personnel to buffer an unpredictable supply chain and resource limitations. Notably, in 1995 during the Ecuador–Peru Alto Cenepa War, leaders from both El Oro and Tumbes maintained this local cross-border support network, taking great risks to transport materials needed for malaria control. Health workers risked their lives and being detained for treason in order to continue sharing epidemiologic information, vector control resources, and medications across the border. These activities were conducted in secret from the national governments. They shared resources across the border because it was the best way to achieve a mutual goal: preventing further malaria transmission, both within the militarized zone and out to other parts of both countries. The collaboration also resulted in a greater exchange of relevant epidemiological information, including maps of *Anopheline* habitats in rural communities on the border that were updated and shared regularly. Health personnel were able to quickly communicate the presence of new malaria cases in the border region and to coordinate targeted interventions, such as cleanup and vector control campaigns in the border canal (demonstrated in photos in Fig. 6). At the same time, malaria control leaders in Ecuador worked to create a robust network of more than 140 public and private diagnostic laboratories, a highly effective and innovative private–public partnership for surveillance and case management.

Experts reported the benefits and challenges of sharing resources to implement binational interventions due to differences in national policies. In 2008 epidemiology personnel from Tumbes conducted a study of 67 individuals infected with malaria in the previous 60 days and tested their close relations for malaria infections. The results of that study revealed that 28% of the family members, 58% of friends living within the same neighbourhood, and 39% of co-workers also had malaria within the following 60 days. Cases were confirmed in the town of Aguas Verdes on the border with Huaquillas, Ecuador. The findings of this study highlighted the necessity of developing a proactive case finding strategy to interrupt the chain of transmission.

Thus in 2009, when 1565 cases of *P. vivax* broke out in a rural border town of Aguas Verdes, in Tumbes (near the town of Chacras, Ecuador), leaders from El Oro and Tumbes worked together to develop and implement a radical malaria control strategy. They planned a focal intervention of mass anti-malarial treatment, an intervention shown to be successful in areas of low transmission, where infections are clustered together in hotspots due to a combination of social and environmental factors [18–21]. In a small pilot intervention, they administered 2000 treatments to the villagers near Chacras, quickly controlling the outbreak. The national level government in Ecuador unofficially supported this intervention, recognizing the expertise of the local team and the need to modify standard procedures in an emergency situation. In light of this success, MoH officials in Tumbes proposed a similar intervention for the nearby localities of Aguas Verdes and Zarumilla, which accounted for 60% of malaria cases in the Tumbes Region in 2008. In a show of solidarity, the Ecuadorian SNEM provided 20,000 anti-malarial treatments to Tumbes. However, the top MoH officials in Tumbes halted the intervention, since it deviated from the national policies. Instead, they opted for a focal intervention (reactive treatment) via active case detection and contact tracing. The strategy consisted of full treatment administration to all family members, friends and coworkers of confirmed malaria cases, even if asymptomatic. Every malaria case that was detected passively was followed-up within the first 24 h and treated with chloroquine (25 mg/kg, total dose over 72 h) plus primaquine (0.5 mg/kg taken for 7 days) to each household contact, excluding elders, pregnant women, and chronically-ill individuals. Officials report that residents of the communities were very accepting and supportive of this intervention, especially given the high burden of disease. This intervention was highly successful in stopping transmission, and highlighted the benefits and challenges of translating national policies from two countries into realistic and effective local disease management in a border setting.

Tumbes was able to reduce the malaria burden in subsequent years by continuing the active case detection strategy. The MoH reported the last case of malaria in Aguas-Verdes and Zarumilla in August of 2010. In 2011 the MoH scaled up this intervention strategy to the entire region of Tumbes, reducing the cases from 672 in 2011 to 83 in 2012, with the last case of malaria reported in the region in November 2012. Officials in Tumbes attribute the continued success of the strategy to the consistent exchange of case information and resources across the border with their counterparts in Ecuador.

### Support from regional malaria control partnerships

Other important regional partnerships supported malaria control in the border region including RAVREDA and the Malaria Control in Border Zones of the Andean Region Programme (PAMAFRO) of the Andean Health Organization (ORAS). RAVREDA began in 2001 through collaboration with the Amazon Malaria Initiative (AMI) of the United States Agency for International Development (USAID) and the Roll Back Malaria Partnership of the WHO/PAHO. The network has aimed to improve surveillance of anti-malarial drug resistance in the Amazonian region, and to address issues of limited diagnostics, inadequate anti-malarial treatment capacity and non-standardized and disintegrated vector control measures in the region [16]. The network’s membership today includes Bolivia, Brazil, Colombia, Ecuador, Guyana, Peru, and Suriname. Machala, the capital city of El Oro province, was designated one of the RAVREDA Ecuador sentinel sites. Both Ecuador and Peru are also part of the PAMAFRO programme of ORAS along with Colombia and Venezuela. PAMAFRO aimed to achieve a 50% reduction of malaria incidence and 70% reduction in malaria mortality in border regions by 2010 [22]. In 2010, Peru was the only country to have reached the target [23] although programme evaluation has proven challenging due to inadequate documentation [22].

### Current malaria control

Through this effective collaboration, the MoH in Ecuador and Peru have successfully suppressed malaria transmission, leading to the elimination of malaria in El Oro in 2011 and in Tumbes in 2012. Today, in Ecuador, malaria diagnostics and anti-malarial treatment are available free of charge for all ages. All febrile individuals who attend MoH clinics are tested for malaria; all pregnant women are tested at the first prenatal checkup and anytime they are febrile. Diagnostics by blood smears are conducted through an extensive network of MoH microscopists at local clinics and hospitals. Additionally, community health volunteers have been trained to collect blood smears from febrile people in their communities. Since 2005, cases of *P. falciparum* are treated using artemisinin-based combination therapy (ACT) and *P. vivax* cases are treated using chloroquine or primaquine. Malaria cases are reported by the nine regional zones of the national Epidemiological Surveillance Subsystem of the MoH (SIVEAlerta). Active foci and case investigations are also carried out; detected cases are defined as indigenous, imported (disease acquired from outside a given area), introduced (transmission from an imported case), induced (through artificial means, such as blood transfusion), and cryptic (no likely mode of transmission exists) [24]. In 2001 SIVE was expanded to a five-part tiered surveillance system, with the top “Alert” subsystem dedicated to health emergency events, including malaria prevention and outbreak response [25]. Vector control includes mapping and elimination of larval habitats, and control of adult mosquitoes by indoor residual spraying (IRS), as well as provision of ITNs to all at-risk populations free of charge. The Ecuadorian government contributes the majority of financing for malaria control in the country, which totals over $2 million per year [14].

Peru is currently in the control phase of malaria management, as designated by the WHO. ITNs and treatment, including ACT, are free for all citizens, and mass diagnostic screening is conducted. Standard operating procedures and policies are in place for malaria surveillance and outbreak investigation and carried out by the Malaria Control Programme, similar to the SNEM in Ecuador [26, 27]. As in Ecuador, surveillance includes both passive and active case detection. Despite these efforts, at the national level Peru had difficulty reducing the malaria burden to the same extent as Ecuador and other nearby countries [28]. In response, the Peruvian government has dramatically scaled up malaria control expenditures since 2011, from $3.6 million in 2005 to $120 million in 2012 [29, 30].

In summary, the following interventions were key strategies in eliminating malaria transmission in the region, according to local experts and the WHO World Malaria Report 2013:

- A comprehensive microscopy network with public and private clinics in high-risk geographic areas.
- Improved quality of microscopy diagnostics.
- Enhanced availability and management policies for anti-malarial treatment.
- Comprehensive and standardized treatment, monitoring, and follow-up of 100% of lab confirmed cases.
- Proactive detection of febrile individuals at the community level through a network of volunteer community health workers.
- Strategic targeting of interventions to patients at increased risk, including pregnant women and children under 5 years old.
- Heightened IRS activities.
- Provision of ITNs to all at-risk populations.
- Mandatory case reporting from the private sector.
- Augmented epidemiologic surveillance system dedicated to malaria (SIVE-Alerta).

## Discussion

Through a binational collaboration over the last 20 years, public health practitioners have eliminated malaria at the Ecuador–Peru border. The success of the programme depended on the local practitioner’s ability to network, to think creatively, to be adaptable, and to work towards a collective goal of improving the health of the people. The resulting trust and openness allowed the public health sector to mobilize rapidly and effectively. The team energized itself when faced with seemingly impossible obstacles, such as the 1995 Cenepa military conflict, and a severe lack of resources. Comprehensive case management, strong and stable leadership, the ability to partner with private and public entities at the local level, thoughtful allocation of resources, and meaningful community engagement were keys to the success of the programmes. This case study reinforces the importance of local, inter-institutional collaborations and horizontal networks to generate effective, sustainable change, especially in conditions of limited or unstable resources and personnel.

One of the keys to the success of this collaboration was recognition and support from the national level MoH and regional networks, which provided external guidance, resources, and legitimacy. Through such multi-level partnerships, both countries benefited from the timely sharing of resources and epidemiological information, and operational research to support novel interventions. Other studies in southern Africa and the Asian Pacific have shown that cooperation among neighbouring nations strengthens individual malaria control initiatives [31, 32]. This type of integrated framework is recommended by PAHO and others [33].

The lessons learned from this collaboration are relevant given the resurgence of mosquito-borne illness in the region. In 2015, other regions of Ecuador reported a resurgence in malaria cases (558 cases were reported in the country in 2015, up from 248 cases in all of 2014 [34]). The region also faces a public health crisis caused by *Aedes aegypti* transmitted febrile illnesses, including dengue fever, chikungunya and zika fever. Over the last two decades, dengue fever (DENV serotypes 1–4) has replaced malaria as the principal cause of vector-borne febrile illness in previously malarial coastal regions in Ecuador [35]. Over a 5-year period (2010–2014), 72,060 cases of dengue were reported in Ecuador (annual average of 14,412 cases). The first cases of autochthonous chikungunya cases were reported in Ecuador at the end of 2014, resulting in a major epidemic in 2015, with over 33,000 cases reported. The first cases of zika were confirmed in Ecuador on January 7, 2016, and currently (06 Oct 2016) 2695 suspected cases of zika have been reported [36]. Both chikungunya and zika have been reported in the border region. Effective control strategies are desperately needed to reduce the burden of disease.

Disease transmission in border regions is notoriously difficult to manage, due to the legal and illegal movement of people and goods, border conflicts, cultural differences, and differences in national public health regulations. This case study highlights the critical importance of supporting local public health leaders who understand the context and the nuances of their local population, and who are invested in long-term collaborations. In many malaria-eliminating countries, the most difficult to reach populations are migrant populations, often men who are geographically clustered in border regions. In these countries, studies have documented a demographic shift from high malaria incidence in women and children to high incidence in working men [31]. In Suriname, for example, transmission risk has shifted from the stabile village populations to the mobile gold mining communities, especially those located on the border with French Guiana. Control strategies in Suriname have led to a substantial decline of malaria transmission in the border region; however, it is recognized that a binational initiative between the countries is needed [37]. Bhutan has seen much success in reducing malaria burden, but one of its greatest challenges lies in the porous border it shares with India, where groups of migrant workers regularly enter the country and reintroduce disease. The country’s elimination strategy is now focused on the establishment of malaria screening centers on the border with existing security checkpoints, and targeting treated bed net distribution to migrant workers as well as at-risk Bhutanese working in the border region [38]. Indeed in 2014 the majority of malaria cases in Ecuador were among men between the ages of 20 and 49 [39].

This case study also highlights the importance of considering social-ecological factors, such as climate events, livelihoods (e.g., agriculture), and construction of large-scale infrastructure (e.g., reservoirs) to understand malaria transmission. Increasing rice production in the region may have increased the risk of malaria transmission. Low-income farming communities, including those associated with rice farming, have historically been at high-risk of malaria [40, 41]. Experts reported anecdotal evidence that the Tahuin Reservoir changed the patterns of malaria transmission in the region. Previous studies in northern coastal Peru showed that malaria incidence was five-times higher in villages with homes built closer to water bodies and irrigation sources [42]. The shift from seasonal to perennial transmission and to hyper-endemicity, has been documented in other areas, such as the Senegal River Basin [43], highlighting the importance of considering the effects of development projects on local disease ecology and microclimate.

It is possible that control efforts in the region were so successful following the 1997–1998 El Niño event, because the climate conditions were averse to malaria propagation. The colder, drier La Niña event from 1998 to 2001 (Fig. 2) may have suppressed transmission, allowing malaria control efforts to push transmission below a critical threshold, thus suppressing further transmission. Prior studies found that malaria and dengue fever epidemics in this region have been associated with El Niño climate events, that caused an increase in local rainfall and air temperatures [44–47]. This is an interesting case where intensified malaria control combined with favourable climate conditions created a scenario for malaria elimination. This highlights the importance of scaling up interventions during periods of low malaria risk (e.g., cool, dry conditions). Future studies should assess the relative effects of climate and malaria control, to disentangle how much of the suppression is truly due to climate versus interventions, and to better inform programme evaluations. It should be noted that climate change projections indicate that strong El Niño events are likely to become more frequent in the future, potentially increasing the risk of malaria epidemics in the region [48, 49].

The experiences in the border region present a cautionary note. There is significant potential for the re-emergence of malaria if surveillance resources are reduced, as was observed in the 1980s and early 1990s. A systematic review of malaria resurgence events worldwide by Cohen and colleagues [50] indicated that Ecuador experienced a resurgence event from 1980 to 1990 due to weakening of control activities, including funding and resource restraints, purposeful cessation and administrative problems, resulting in a subsequent loss in public confidence in SNEM in the early 1980s [51–53]. Other studies indicated that malaria resurgence in Latin America was strongly associated with the weakening IRS operations [53] and anti-malarial drug resistance. Today, the Ecuadorian government contributes the majority of financing for malaria control in the country. Since 2007, it has reduced spending on malaria control programmes by 75%, from over $8 million in 2007 to $2 million in 2012 [14]. The Peruvian government has dramatically scaled up spending on malaria control since 2011, from $3.6 million in 2005 to $120 million in 2012 [29, 30].

In 2015 the vector control service in Ecuador became decentralized and reorganized under a new, horizontal model of vector control, similar to Peru. The aim is to better integrate vector control activities within the national Health Services framework, but there is the risk that with these large structural changes may come an initial loss of operational capacity [54]. While it should not be assumed that any of these structural changes will lead to a resurgence of malaria, it is important to consider that resurgence events have occurred here and elsewhere when a weakening of control activities coincided with social-ecological and political factors (e.g., El Niño event, human migration, reduction in global oil prices leading to decreased national revenue and MoH budget in Ecuador). Given this transition, it would be beneficial for the MoH in both countries to conduct ongoing evaluations of control programmes, as was done recently in Guayas, Ecuador [55], to ensure ready response to outbreaks and optimal allocation of resources [20].

In this study, the perspectives of a small group of local public health leaders are presented. The majority of the activities and events described were triangulated using other documented evidence. This group was recommended by their peers as being the most knowledgeable to recount the true facts that took place on the ground during malaria control efforts, and they held a range of positions within the MoH, including decision-makers and field personnel. Future reporting on this topic could benefit from the viewpoint of the national MoH and other government stakeholders, both current and those who were in office during the elimination period, in order to obtain a clear understanding of the national context. In addition, it would be important to include other key local stakeholders, such as community leaders and the private sector.

## Conclusions

Through the results of this study and the experience of experts working in the region, the following key recommendations and lessons learned have been identified:

- Consider radical, non-traditional interventions when necessary.
- Explore alternative techniques using small, localized pilot projects. The evidence generated can impact national policy.
- National policies and standards need to be translated to local realities.
- An active case-finding surveillance system coupled with a strict treatment plan can be translated to almost any environment.
- A small team of dedicated, long-term public health practitioners can be nimble and effective when empowered by regional and national networks.
- It is important to share experiences with the next generation of public health practitioners so that they too can learn from these lessons and not repeat the mistakes of the past.

## Additional file

**Additional file 1: Table S1.** Table of key informant experts in the study and their position during the elimination period.

## Abbreviations

ACT: artemisinin-based combination therapy
AMI: Amazon Malaria Initiative
CDC: U.S. Centers for Disease Control
INAMHI: National Institute of Meteorology and Hydrology (Ecuador)
INEC: National Institute of Census and Statistics (Ecuador)
IRS: indoor residual spraying
ITN: insecticide treated bed net
MoH: Ministry of Health
NOAA: National Oceanic and Atmospheric Administration
ONI: Oceanic Niño Index
ORAS: Andean Health Organization
PAHO: Pan American Health Organization
PAMAFRO: Malaria Control in Border Zones of The Andean Region
RAVREDA: Amazon Network for the Surveillance of Antimalarial Drug Resistance
SENAMHI: National Institutes of Meteorology and Hydrology (Peru)
SIVE: Epidemiological Surveillance Subsystem (Ecuador)
SNEM: National Vector Control Service
USAID: United States Agency for International Development
