# Supplementary material for: Successful malaria elimination in the Ecuador–Peru border region: epidemiology and lessons learned
Source: Malar J. 2016 Nov 28;15:573. doi: 10.1186/s12936-016-1630-x (PMC5126842; doi:10.1186/s12936-016-1630-x)
Supplement: Supplementary file 1 — Additional file 1: Table S1. Table of key informant experts in the study and their position during the elimination period. [file 12936_2016_1630_MOESM1_ESM.docx]

Supplement 1**.** Table of key informant experts in the study and their position during the elimination period:

|  | **Name** | **Position** |
| --- | --- | --- |
| **El Oro** | Dr. Efraín Beltrán Ayala | Chief Coordinator SNEM Zone 8 |
|  | Dr. Mercy Silva | Director of SNEM Zone 8 Entomology Laboratory and Diagnostics Network |
|  | Lic. Tania Ordoñez | Director of Community Engagement and Surveillance |
|  | Dr. Ana Arichabala | Coordinator of Health Area 1 |
|  | Lic. Patricia Navarette | Epidemiologist Health Area 1 |
|  | Dr. Mariano Ambuludi | Director SNEM Zone 8 |
|  | Dr. Marcia Veintimilla | Coordinator Brisas del Mar health clinic |
|  | Dr. Álvaro Calle | Epidemiologist SNEM Zone 8 |
| **Tumbes** | Dr. Fernando Quintana | Chief Epidemiologist |
|  | Tec. Vieto Sacerdoval Velante | Entomologist |
|  | Tec. Carlos Eladio Olivares Barrientos | Regional Coordinator of Surveillance MINSA Region of Tumbes |
|  | Blgo. Ricardo Alvaro Aradivel | Laboratory Technician |
|  | Tec. Victor S. Rios Verasteque | Field Technician, Surveillance and Vector Control |
